# Ancient administrative handwritten documents: X-ray analysis and imaging

**DOI:** 10.1107/S1600577515000314

**Published:** 2015-01-30

**Authors:** F. Albertin, A. Astolfo, M. Stampanoni, Eva Peccenini, Y. Hwu, F. Kaplan, G. Margaritondo

**Affiliations:** a Faculté des Sciences de Base, Ecole Polytechnique Fédérale de Lausanne (EPFL), CH-1015 Lausanne, Switzerland; b Swiss Light Source, Paul Scherrer Institut (PSI), Villigen, Switzerland; c Institute for Biomedical Engineering, ETHZ, Zürich, Switzerland; d Department of Physics and Earth Sciences, University of Ferrara, Italy; e Laboratory TekneHub, Technopole of Ferrara, Italy; f Institute of Physics, Academia Sinica, Taipei, Taiwan; g Laboratoire d’Humanités Digitales, Ecole Polytechnique Fédérale de Lausanne (EPFL), Switzerland

**Keywords:** cultural heritage, ancient manuscripts, ancient inks, X-ray fluorescence, phase contrast, refractive index imaging, differential phase contrast

## Abstract

Handwritten characters in administrative antique documents from three centuries have been detected using different synchrotron X-ray imaging techniques. Heavy elements in ancient inks, present even for everyday administrative manuscripts as shown by X-ray fluorescence spectra, produce attenuation contrast. In most cases the image quality is good enough for tomography reconstruction in view of future applications to virtual page-by-page ‘reading’. When attenuation is too low, differential phase contrast imaging can reveal the characters from refractive index effects. The results are potentially important for new information harvesting strategies, for example from the huge Archivio di Stato collection, objective of the Venice Time Machine project.

## Abstract 2

The heavy-element content of ink in ancient administrative documents makes it possible to detect the characters with different synchrotron imaging techniques, based on attenuation or refraction. This is the first step in the direction of non-interactive virtual X-ray reading.

## 1. Reading ancient handwritings: role of X-rays   

We have correlated the chemical analysis by X-ray fluorescence spectroscopy (XRF, with a portable instrument) of 15th to 17th century administrative Italian documents and the X-ray imaging of the handwritten characters using synchrotron radiation. This combined approach has enabled us to extensively and flexibly characterize the ink composition prior to the imaging tests, then to exploit the properties of synchrotron radiation for advanced X-ray imaging (including refraction-based contrast when attenuation is weak) and tomography, and finally to correlate the results.

Heavy elements in inks (Del Carmine *et al.*, 1996 ▸) normally allow their detection by X-ray attenuation. However, strong concentration fluctuations occur between different manuscripts and between different areas of the same specimen. Sometimes low concentration impedes detection by attenuation; an alternative method is refractive-index contrast (Hwu *et al.*, 1999 ▸) in differential phase contrast imaging (Weitkamp *et al.*, 2005 ▸).

Our study is part of the international Venice Time Machine (VTM) project (http://dhvenice.eu). The Archivio di Stato in Venice holds about 80 km of archival documents spanning over ten centuries and documenting every aspect of the Venetian Mediterranean Empire. If unlocked and transformed into a digital data system, this information could change significantly our understanding of European history. But the sheer mass of data is a problem. VTM plans to digitalize and decipher the entire collection in 10–20 years.

To facilitate and accelerate this task, the project also explores new ways to virtually ‘read’ manuscripts, rapidly and non-invasively. We specifically plan to use X-ray tomography to computer-extract page-by-page information from sets of projection images. The raw data can be obtained without opening or manipulating the manuscripts, reducing the risk of damage and speeding up the process.

This approach is based on precursor projects exploiting X-rays to decipher documents. Notably, synchrotron light was used to retrieve ‘lost’ text from the ‘Archimedes Palimpsest’ (Bergmann, 2000 ▸) with X-ray fluorescence. The use of X-ray tomography to analyze handwriting was pioneered by Seales *et al.* (Lin & Seales, 2005 ▸; Baumann *et al.*, 2008 ▸; Seales *et al.*, 2011 ▸) with the EDUCE project. A top-level program in this direction was launched by T. Wess of Cardiff University (Mills *et al.*, 2012 ▸; Patten *et al.*, 2013 ▸). In particular, the project virtually ‘unrolled’ scrolls producing flat readable images and assessed the possibility of damage by X-ray exposure.

These efforts are part of several pioneering studies exploiting synchrotron techniques for the humanities and art (Janssens, 2011 ▸; Creagh & Bradley, 2007 ▸; Caforio *et al.*, 2014 ▸; Morigi *et al.*, 2010 ▸; Reischig *et al.*, 2009 ▸; Dik *et al.*, 2008 ▸, 2010 ▸; Možir *et al.*, 2012 ▸; Faubel *et al.*, 2007 ▸; Lucarelli & Mandò, 1996 ▸; Kennedy *et al.*, 2004 ▸; Gunneweg *et al.*, 2010 ▸; Murphy *et al.*, 2010 ▸). In general, such efforts have demonstrated high effectiveness in clarifying issues, notably those related to chemical and microstructural properties and their relations to issues such as environment-related damage, origin, dating *etc.* However, they still realise only a small fraction of the potential applications of synchrotron experiments in these domains.

Along the path to deciphering the Venice collection, we must deal with a series of key issues. A fundamental one is the nature of the ink in everyday documents (as opposed to pieces of high artistic or historical value) and its detection by X-rays. This is the subject of our present study.

The issue is by no means clear *a priori*. The very nature of the VTM project requires studying archival documents such as ship records, notary papers, work contracts, tax declarations, commercial transactions and demographic accounts. For such items, the ink composition is scarcely documented. Furthermore, the Archivio di Stato collection spans ten centuries, with inevitable fluctuations in the ink chemistry.

Clarifying these issues has a remarkable and multi-faceted potential impact. First, in addition to the Venice collection the techniques could be applied to many documents at risk throughout the world. Second, the chemical and microstructural information is also relevant for the deterioration mechanisms and could help in preventing them.

Our main results are the following. First, heavy elements are systematically detected in the inks of personal and commercial documents over the three centuries investigated here. Second, the chemical composition is basically consistent with the historical records of the inks (Yale University Library Special Collections Conservator Unit, 2012 ▸; Capella, 420 ▸). Third, the relative amounts of heavy elements change drastically between different document areas and from manuscript to manuscript, with no historical trend. Fourth, there is, as expected, a direct correlation between the quality of attenuation-contrast images and the chemical composition.

In the best situations the X-ray attenuation image quality is good enough to perform tomographic reconstruction of phantom ‘volumes’ created by stacking ancient manuscript fragments. In the worst cases, even simple visualization is a problem.

In such cases, the properties of synchrotron light become very helpful. Indeed we exploited its spatial coherence (Margaritondo, 2002 ▸) to record images with refractive-index contrast. As demonstrated by the extensive experience (Hwu *et al.*, 1999 ▸) with other types of specimens, this could allow the recognition of faded-out or very weak characters.

## 2. Chemistry of ancient inks in everyday administrative writings   

Why could heavy elements be present in ancient inks? Let us start from black inks (Yale University Library Special Collections Conservator Unit, 2012 ▸; Capella, 420 ▸). For many centuries, Europe widely used a formula generically denominated as ‘iron gall’, a name suggesting the presence of iron. A less common black ink, with no heavy elements, was the Roman atramentum scriptorium, based on lampblack with a gum binder.

In addition to black inks, part of the documents, typically those with important writings, used coloured inks. Some of them contained heavy elements, *e.g.* mercury in cinnabar red (Delaney *et al.*, 2014 ▸). Other common recipes did not use heavy elements, *e.g.* the Brazil red (Yale University Library Special Collections Conservator Unit, 2012 ▸).

Even the generic iron gall formula corresponded to a wide variety of ingredients and recipes (Yale University Library Special Collections Conservator Unit, 2012 ▸). The basic fabrication process was the reaction of an acid with an iron compound. The most common procedure involved tannic acid (C_76_H_52_O_46_) and iron sulfate (FeSO_4_) in rainwater, white wine or vinegar (Smith, 2009 ▸).

Tannic acid was obtained from plants, the richest source being the ‘galls’ produced by trees in response to parasite attacks (*e.g.* by gall wasps); for example, the British oak galls or the top-quality ‘Aleppo galls’. Iron sulfate was known as ‘green vitriol’, extracted from mines, notably coal mines. The reaction of tannic acid with FeSO_4_ produced, with oxygen exposure, ferrotannate, a black pigment.

In addition to the pigment, the black inks also contained a water-soluble binder. One of the most common was gum arabic. This is a natural product of trees, *e.g.* acacia, rich in polysaccharides and glycoproteins; its main component is arabin (Smith, 2009 ▸; Ruggiero, 2002 ▸), the calcium salt of the polysaccharide arabic acid. Other ingredients could be present, such as logwood pigment.

One important property of iron gall inks is their time evolution. Although a small quantity of black pigment is developed with the oxygen present in the solution, most is produced with atmospheric oxygen after writing, over hours or days.

Furthermore, the ink is corrosive over very long periods of time, chemically attacking the substrate. The ink–substrate interaction was extensively analyzed by Banik *et al.* (1983 ▸); and Neevel & Reissland (1997 ▸), Proost *et al.* (2004 ▸) and Kanngiesser *et al.* (2004 ▸) attacked this issue with synchrotron radiation XANES (X-ray absorption near-edge structure) and microfluorescence techniques.

The above features agree with our chemical analysis, based on XRF. We performed the XRF experiments with a portable µ-XRF spectrometer ARTAX (model 200; Brucker). This instrument uses an air-cooled fine-focus Mo X-ray source with a collimator. The detector is a Peltier cooled silicon drift device with 10 mm^2^ active area, reaching a resolution better than 150 eV for the Mn *K*α fluorescence, with a count rate up to 10^5^ s^−1^ and a dead time <10% at 4 × 10^4^ s^−1^. The instrument is equipped with a visible-light CCD camera (20× magnification) and with a pointing laser, to identify and picture the analyzed area. In our tests, XRF spectra were recorded under He flux, operating the spectrometer at 15 kV and 1500 µA (exposure time 180 s).

The specimens were carefully handled to avoid contamination but posed no particular fragility problems. The portable instrument enabled us to analyze whole manuscripts and several micro-areas of each manuscript. Its performances were perfectly adequate to our chemical analysis without requiring a synchrotron source. In principle, the XRF instrument could also yield images, but the corresponding time per image would have been exceedingly long (several days per picture) for our final objectives, and would exceedingly complicate tomography.

**Figure 1:**
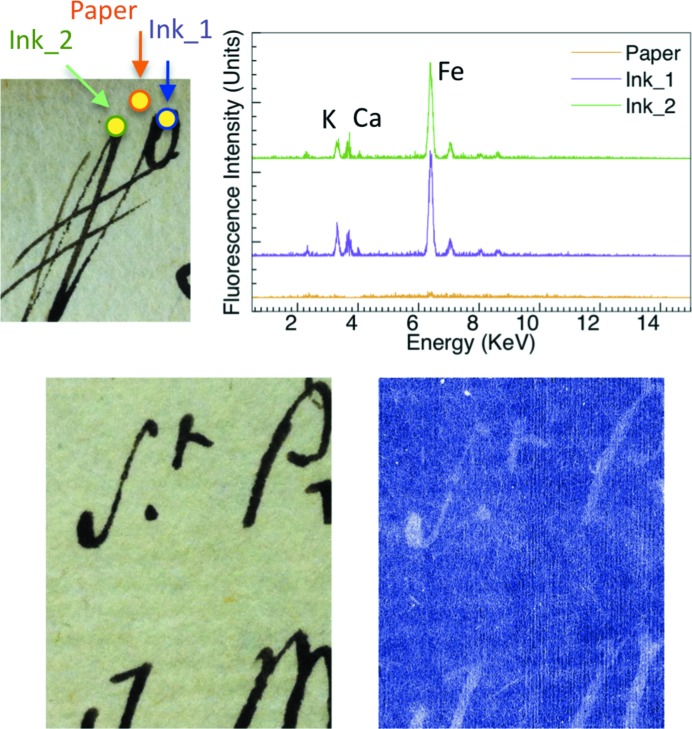
Results for a 1590 Italian (Tuscany) handwritten record. Top right: X-ray fluorescence spectra taken from the 200 µm-wide spots marked on the top-left visible picture. Bottom: comparison of a 14 mm × 18 mm visible picture (left) with an X-ray image taken in the free propagation mode of the TOMCAT beamline (15 keV, exposure time 80 ms) (Stampanoni *et al.*, 2007 ▸). In this acquisition mode, two X-ray effects are visible: attenuation allows character recognition whereas phase contrast enhances the paper fibres.

Figs. 1 ▸ ▸ ▸–4 ▸ include XRF spectra taken from 200 µm-wide (the collimator size) spots of 16th and 17th century Italian writings of personal and administrative nature and from a 15th century religious parchment manuscript. We acquired them using the same geometric conditions for all specimens.

All spectra revealed Fe in the ink areas, as opposed to the substrate areas. In addition, we detected Ca in the same ink areas. This is consistent with the use of gum arabic as the binder. Calcium was also found in the substrate of the parchment specimens presumably due to chalk used during support preparation (Van der Snickt *et al.*, 2008 ▸).

These rather straightforward results are accompanied by several other findings. In the 1590 (Fig. 1 ▸) and 1664 (Fig. 3 ▸) specimens the ink also contains Cu and Zn, which are known to contribute to the ink–substrate interaction (Banik *et al.*, 1981 ▸). Furthermore, the Fe and Ca content varied substantially. We reached this semi-quantitative conclusion using spectra recorded with geometric, voltage and current conditions as similar as possible for different specimens and areas.

The different amounts of Fe correlate well with the X-ray attenuation contrast, as shown in Figs. 1 ▸–4 ▸. These figures include visible photographs as well as X-ray attenuation images.

The radiographs were taken at the TOMCAT beamline (Stampanoni *et al.*, 2007 ▸) of the Swiss Light Source, Paul Scherrer Institute. The X-ray source was a 2.9 T superbending magnet with a critical photon energy of 11.1 keV, and an electron beam (source) size of 46 µm × 16 µm. The main optical component is a fixed-exit double-crystal multilayer monochromator that covers the energy range 6–45 keV. The crystal optics is mounted on two independent high-precision goniometers. The first crystal has motorized pitch, roll and horizontal translation; the second crystal has the same degrees of freedom and, in addition, yaw and vertical translation. The entire system is positioned on a base plate that can be vertically adjusted. The vertical size of the beam is ‘controlled’ by moving the end-station along the beam path (up to 15 m travel range).

For the images of Figs. 1 ▸–3 ▸, the photon energy was 15 keV, and 25 keV for Fig. 4 ▸. Throughout the image-recording experiments the storage ring current was kept constant at 400 mA by operation in the top-up mode.

TOMCAT can record images with different modes, described in detail by Stampanoni *et al.* (2007 ▸). The images in Figs. 1 ▸–3 ▸ were taken with the free-propagation operation mode and reflect both phase contrast and attenuation contrast. Phase contrast is primarily visible in the microscopic substrate features. Attenuation contrast prevails in the ink areas.

**Figure 2:**
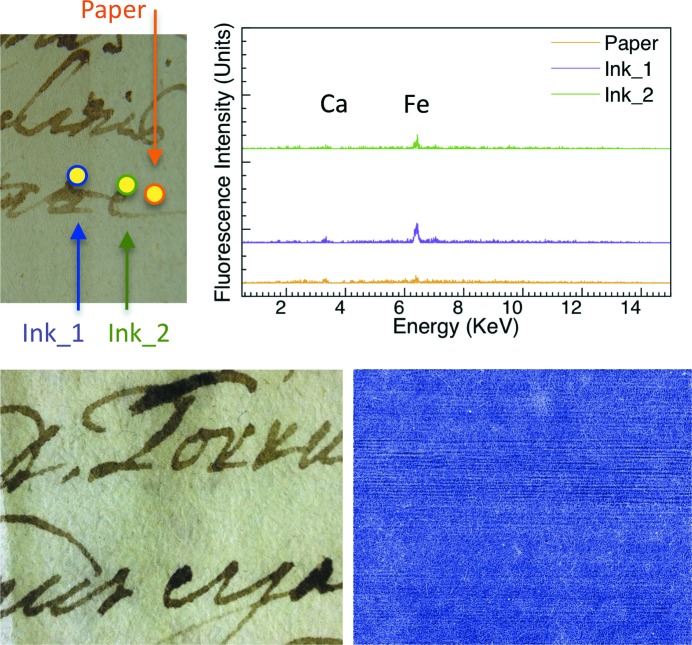
Results like those of Fig. 1 ▸, for a 1646 Italian (Tuscany) specimen (image size 18 mm × 14 mm). Here, the iron concentration in the ink is too low to detect characters with X-ray attenuation whereas the paper fibres are still visible by phase contrast.

**Figure 3:**
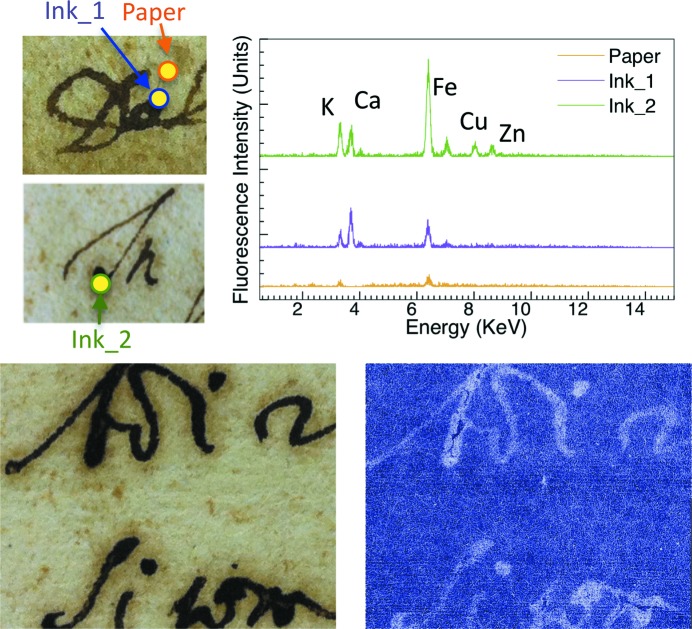
Results like those of Fig. 1 ▸, for a 1664 Italian (Tuscany) specimen (image size 18 mm × 14 mm). The radiograph reveals otherwise nearly invisible holes caused by ink-induced paper corrosion.

The effects of iron on X-ray attenuation contrast are visible when comparing Fig. 1 ▸ and Fig. 3 ▸ with Fig. 2 ▸: in the 1646 specimen the Fe peak is rather weak and the X-ray image shows no clear evidence of characters.

**Figure 4:**
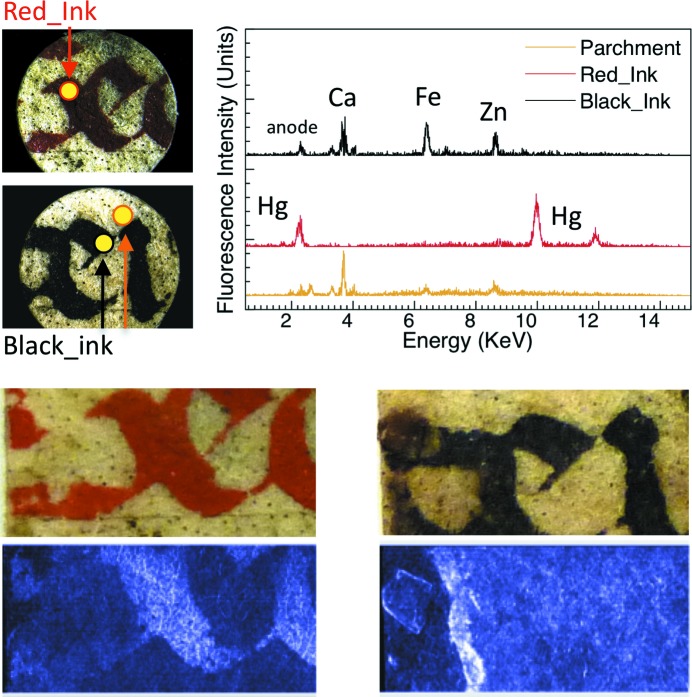
Results similar to those of Fig. 1 ▸, for 15th century religious writing on parchment, of Italian origin (image size 18 mm × 7 mm). The bottom part shows a comparison of two visible pictures of red and black ink characters and two X-ray absorption images below (recorded with a grating interferometer at TOMCAT, DPC mode, 25 keV, exposure time 80 ms) (Stampanoni *et al.*, 2007 ▸).

In addition to black (iron gall) ink, ancient manuscripts could also have colour inks. Our 15th century parchment specimen included red characters, written with ink containing Hg (cinnabar). Hg was indeed detected in the X-ray fluorescence spectra, as seen in Fig. 4 ▸, and produced excellent attenuation contrast. On the contrary, the Fe signal from the black characters is weak, and the contrast low.

**Figure 5:**
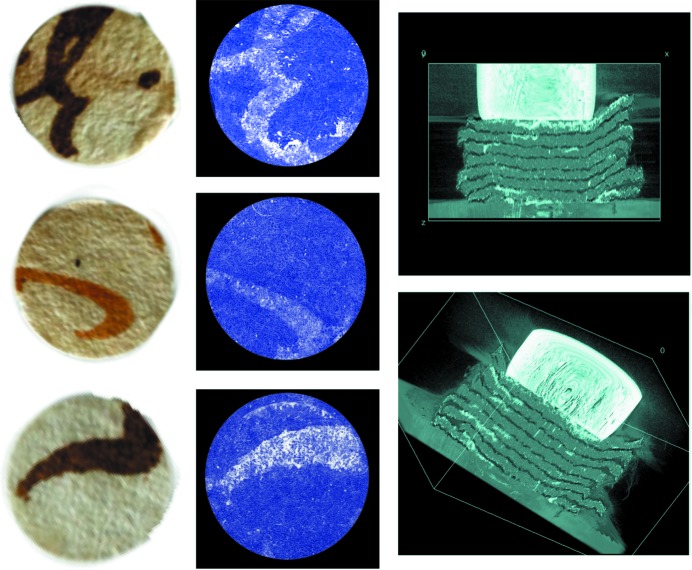
Tomography results for a 1679 Italian (Tuscany) document. Centre: three reconstructed images of virtual ‘pages’ with different characters, corresponding to the visible photographs on the left-hand side. Right: three-dimensional reconstructions showing side views of the pages with ink visible over them. The tomography was performed for a stack of eight manuscript fragments simulating a small volume. The bright area in the two right-hand-side images is a small magnet keeping the fragment stack in place.

Positive tests of tomographic reconstruction are shown in Fig. 5 ▸ for a 1679 specimen, consisting of a stack of eight 0.8 cm-diameter fragments simulating a small volume. We took a set of projection images over a π rad range, at an angular distance of π/10^3^ from each other, using 15 keV photons and a time exposure of 10 ms per picture.

Fig. 5 ▸ shows three tomographically reconstructed ‘pages’ with recognizable characters (comparable results were obtained for the other pages) and two three-dimensional reconstructed side views. Note that the spatial resolution (6.5 µm detector pixel) is largely sufficient not only to distinguish different ‘pages’ but also to determine whether the writing is on the front or on the back of each ‘page’.

## 3. Alternate imaging modes   

How could we handle the cases of low attenuation contrast? Synchrotron light can provide the answer, allowing imaging techniques with contrast mechanisms related to the imaginary part of the complex refractive index, *i.e.* to phase-related phenomena like refraction (Margaritondo & Hwu, 2013 ▸).

**Figure 6:**
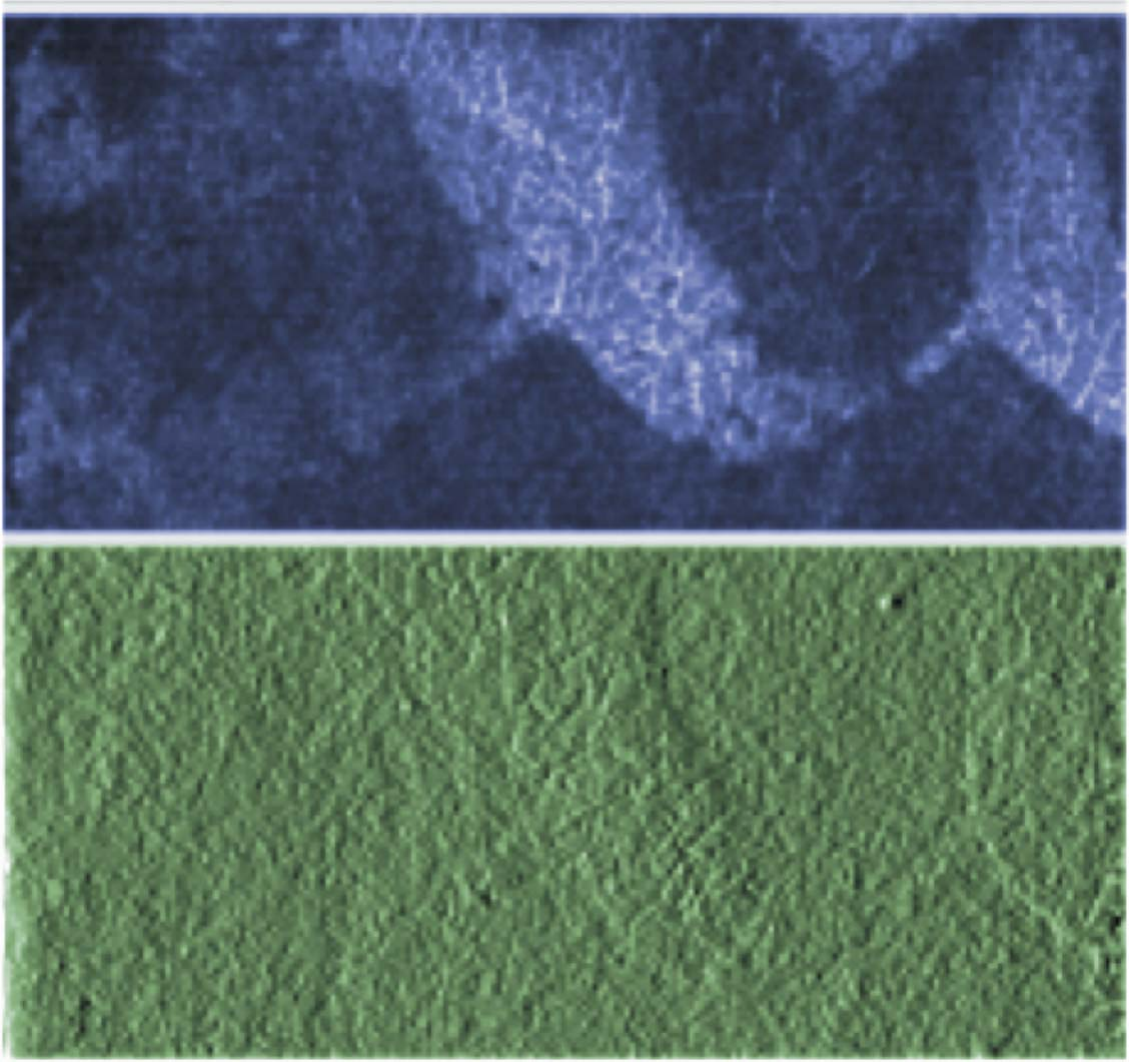
Images recorded in the DPC mode of TOMCAT (Weitkamp *et al.*, 2005 ▸; Stampanoni *et al.*, 2007 ▸) for the red-character area of Fig. 4 ▸. The top picture corresponds to scattering contrast and the bottom to refraction contrast.

Fig. 6 ▸ shows a test: the imaged area is the same as that of the red characters of Fig. 4 ▸, but the contrast mechanism is different. The image pair was recorded using differential phase contrast (DPC), discussed in detail by Weitkamp *et al.* (2005 ▸). With suitable digit-by-digit mathematical processing (Weitkamp *et al.*, 2005 ▸), the raw DPC images yield pictures corresponding to absorption, scattering and refraction. Fig. 4 ▸ shows indeed DPC absorption-contrast images obtained in this way. Fig. 6 ▸ shows instead scattering and refraction images.

The important point here is that the refraction images reflect the local specimen morphology rather than only its chemical composition. We can speculate that what we see in Fig. 6 ▸ reflects the substrate morphology modified by the writing process or by the ink–substrate interaction. This could be used to detect a character when the attenuation contrast gives a faint picture.

## 4. Conclusions and perspectives   

Our tests yielded overall positive results along the path to virtual X-ray reading but stressed some potential problems. The positive point is that the European recipes to fabricate common inks over several centuries produced in most cases heavy elements sufficient for character detection by X-ray attenuation. We verified that the corresponding image quality is suitable for advanced tomographic reconstruction, at least for a limited number of pages.

In some instances, however, the heavy-element content was too weak. Our preliminary tests indicate that these cases can be potentially handled with refraction images.

Note that the attenuation images and the refraction images carry a wealth of information besides the written characters. They can notably reveal information, complementary to other experimental techniques (Proost *et al.*, 2004 ▸; Kanngiesser *et al.*, 2004 ▸), related to the ink–substrate interaction, which in many cases leads to corrosion and deterioration. This will hopefully contribute to the identification of ways to prevent long-term damage.

Results such as those discussed here open the way to a new strategy for information harvesting from ancient documents, alternate to page-by-page recording of visible pictures. A single tomographic set could yield the same information more rapidly and with minimized interaction with the document.

Many problems remain to be solved along this path: in particular, we must test the extension of the tomographic reconstruction to larger-size documents. Also crucial will be the development of adequate software, for example for automatic analysis of reconstructed images. However, the technique must still be optimized to reach its best performances. On a positive side, throughout our tests we found no evidence whatsoever of radiation damage, consistent with Patten *et al.* (2013 ▸).
